# Morphological and functional evolution of gametophytes in epilithic *Hymenasplenium murakami-hatanakae* (Aspleniaceae): The fifth family capable of producing the independent gametophytes

**DOI:** 10.1007/s10265-024-01553-0

**Published:** 2024-06-25

**Authors:** Katsuhiro Yoneoka, Tao Fujiwara, Toshifumi Kataoka, Kiyotaka Hori, Atsushi Ebihara, Noriaki Murakami

**Affiliations:** 1 Makino Herbarium, Tokyo Metropolitan University, 1-1 Minami-Osawa 1-1, Hachioji, Tokyo 192-0397 Japan; 2 The Mt. Fuji Institute for Nature and Biology, Showa University, 4562 Kamiyoshida, Fujiyoshida, Yamanashi, 403-0005 Japan; 3 Makino Botanical Garden, 4200-6 Godaisan, Kouchi-City, Kouchi, 780-0870 Japan; 4 Department of Botany, National Museum of Nature and Science, 4-1-1 Amakubo, Tsukuba, Ibaraki 305-0005 Japan

**Keywords:** Aspleniineae, Cordate gametophyte, East Asia, Epiphyte, Eupolypod II, Ferns, Morphological evolution, Non-cordate gametophyte, Separation of generation

## Abstract

The fern independent gametophytes that can maintain populations by vegetative reproduction without conspecific sporophytes have been considered an unusual phenomenon found in some epiphytic or epilithic species of Hymenophyllaceae, Pteridaceae, Lomariopsidaceae, and Polypodiaceae. By chance, the discovery of mysterious strap-like gametophytes on Izu-Oshima Island, Japan, has led to the hypothesis that *Hymenasplenium murakami-hatanakae*, a fern species belonging to Aspleniaceae, can also form independent gametophytes. Our investigation revealed gametophyte populations of *H. murakami-hatanakae* on three islands in the Izu Islands. Based on chloroplast DNA analysis of the gametophyte and sporophyte populations, the gametophytes were found to be maintained by vegetative reproduction without a new supply of spores from sporophytes. A comparison of the surrounding vegetation at the collection sites showed that environmental factors such as light and humidity may influence the maintenance of gametophyte populations. These results clearly show that *H. murakami-hatanakae* is one of the ferns capable of forming independent gametophytes. This is the first report of independent gametophytes from the suborder Aspleniineae (eupolypod II). The discovery of the independent gametophyte within a phylogenetic lineage previously thought not to form independent gametophytes will provide important insights into the morphological and functional evolution of gametophytes in ferns.

**Supplementary Information:**

The online version contains supplementary material available at 10.1007/s10265-024-01553-0.

## Introduction

Historically, the diversity of fern gametophytes has been overlooked in studies of plant biodiversity. Generally, land plants alternate between two generations with different ploidy levels: haploid gametophytes and diploid sporophytes. Ferns are unique among land plants in that each generation is nutritionally independent of the other (Pinson et al. 2017a). Although the fern life cycle is characterized by separation of generation, most previous studies of fern diversity have focused only on the sporophyte generation (Nayar and Kaur 1971). This bias is due to the small size of gametophytes compared to conspecific sporophytes and the lack of morphological characters for species identification. Consequently, the study of gametophytic diversity has been hampered, and the gametophytic flora has been neglected for many years.

One of the case studies reporting on wild gametophytes focused specifically on independent gametophytes (Farrar 1967). Independent gametophytes are initially defined as fern gametophytes whose sporophytes are completely absent (obligate independence) or extremely rare (facultative independence), and those are maintained only by vegetative reproduction through gemmae, clonal forming or branching from the gametophytes (Farrar 1967; Rumsey and Sheffield 1996). Several physiological and ecological studies suggest that such gametophytes were formed due to their higher environmental tolerance compared to conspecific sporophytes (Ebihara et al. 2019; Farrar 1998; Watkins et al. 2007). In the case of *Vittaria appalachiana* Farrar & Mickel, the most famous example of independent gametophytes because it was recorded first, the sporophytes might have become extinct due to global cooling during the last glacial period, whereas the conspecific gametophytes with stronger cold tolerance survived with gemma reproduction around the Appalachian Mountains of North America for more than 10,000 years (Farrar and Mickel 1991). Such phenomenon, significantly different from the typical life cycle of ferns, was previously thought to be extremely rare and restricted to glaciated regions.

Recent studies of gametophyte diversity using DNA barcoding methods have revealed independent gametophytes not only from glaciated cold regions but also from warm temperate and even tropical areas unrelated to glaciers (Chen et al. 2013; Kuo et al. 2017). The gametophyte populations maintained by vegetative reproduction are discovered both within and beyond the geographic range of the conspecific sporophytes (Duffy et al. 2015; Ebihara et al. 2013; Park et al. 2020). The prominent distribution pattern of gametophyte dominance is intriguing in that it resembles the reproductive strategy of bryophytes rather than ferns (Guillet et al. 2021). It is now clear that independent gametophytes are commonly formed in at least four families: Hymenophyllaceae, Pteridaceae, Lomariopsidaceae, and Polypodiaceae (Table 1). As a result of these discoveries, the definition of independent gametophytes was also changed from the species level to the population level (Kuo et al. 2017), i.e., populations of fern gametophytes maintained by vegetative reproduction alone and distributed completely outside the geographic distribution range of functional conspecific sporophytes were redefined as obligate independence, and those populations maintained by rare spore supply from nearby growing functional conspecific sporophytes were also redefined as facultative independence. The independent gametophytes that have been able to grow for a long time may contribute to sporophytic diversification by providing gametes in all climatic regions on the earth (Ebihara et al. 2009). The discovery of successive independent gametophytes has highlighted the need to further investigate of species diversity within the gametophyte generation. Therefore, elucidation of the species diversity of long-lived fern gametophytes is one of the priority issues in pteridology.

**Table 1:** List of the independent gametophytes in previous and present studies. *Hymenasplenium murakami-hatanakae*, added by this study, is indicated in bold. "*" indicates that it was estimated based on the habitat of sporophytes of related species. Modified from a table in Pinson et al. (2017a)

No	Family	Taxon	Morphological type of gametophyte	Habitat of the con-specific sporophyte	Reference
1	Hymenophyllaceae	*Crepidomanes intricatum* (Farrar) Ebihara & Weakley	Filamentous	epilithic	Ebihara et al. (2008)
2	Hymenophyllaceae	*Vandenboschia kalamocarpa* (Hayata) Ebihara	Filamentous	epilithic	Ebihara et al. (2009)
3	Hymenophyllaceae	*Vandenboschia speciosa *(Willd.) G.Kunkel	Filamentous	epilithic	Rumsey et al. (1990)
4	Hymenophyllaceae	*Vandenboschia cyrtotheca* (Hillebr.) Copel	Filamentous	epilithic	Pinson et al. (2017a, b)
5	Hymenophyllaceae	*Didymoglossum petersii *(A.Gray) Copel	Filamentous	epilithic	Farrar (1990); Pinson et al. (2017a)
6	Hymenophyllaceae	*Hymenophyllum tayloriae* Farrar & Raine	Ribbon-like	epilithic	Raine et al. (1991)
7	Hymenophyllaceae	*Hymenophyllum recurvum* Gaudich	Ribbon-like	epilithic	Pinson et al. (2017a)
8	Hymenophyllaceae	*Hymenophyllum wrightii* Bosch	Ribbon-like	epilithic	Duffy et al. (2015); Park et al. (2020)
9	Hymenophyllaceae	*Hymenophyllum badium* Hook. & Grev	Ribbon-like	epilithic	Ebihara et al. (2013); Park et al. (2020)
10	Hymenophyllaceae	*Hymenophyllum barbatum* (Bosch) Baker	Ribbon-like	epilithic	Ebihara et al. (2013); Park et al. (2020)
11	Hymenophyllaceae	*Hymenophyllum* sp.1	Ribbon-like	epilithic*	Ebihara et al. (2013)
12	Hymenophyllaceae	*Hymenophyllum* sp.2	Ribbon-like	epilithic*	Ebihara et al. (2013)
13	Hymenophyllaceae	*Callistopteris baldwinii *(D.C.Eaton) Copel	Ribbon-like	epilithic	Dassler and Farrar (1997); Pinson et al. (2022)
14	Hymenophyllaceae	*Callistopteris apiifolia *(C.Presl) Copel	Ribbon-like	epilithic	Ebihara et al. (2013): Nitta et al. (2021)
15	Pteridaceae	*Vittaria graminifolia* Kaulf	Ribbon-like	epiphytic/epilithic	Farrar and Landry (1987); Pinson et al. (2017b)
16	Pteridaceae	*Vittaria appalachiana Farrar* & Mickel	Ribbon-like	epiphytic/epilithic*	Farrar (1967); Farrar and Mickel (1991)
17	Pteridaceae	*Antrophyum henryi *Hieron	Ribbon-like	epiphytic/epilithic	Chen et al. (2013)
18	Pteridaceae	*Antrophyum formosanum* Hieron	Ribbon-like	epiphytic/epilithic	Chen et al. (2013)
19	Pteridaceae	*Antrophyum sessilifolium* (Cavanilles) Sprengel	Ribbon-like	epiphytic/epilithic	Chen et al. (2013)
20	Pteridaceae	*Antrophyum parvulum *Blume	Ribbon-like	epiphytic/epilithic	Chen et al. (2013)
21	Pteridaceae	*Antrophyum obovatum *Baker	Ribbon-like	epiphytic/epilithic	Park et al. (2020)
22	Pteridaceae	*Haplopteris heterophylla C*.W.Chen, Y.H.Chang & Yea C.Liu	Ribbon-like	epiphytic/epilithic	Chen et al. (2013)
23	Pteridaceae	*Haplopteris yakushimensis C*.W.Chen & Ebihara	Ribbon-like	epiphytic/epilithic	Kuo et al. (2017)
24	Pteridaceae	*Haplopteris anguste-elongata* (Hayata) E.H.Crane	Ribbon-like	epiphytic/epilithic	Chen et al. (2013)
25	Pteridaceae	*Haplopteris elongata* (Sw.) E.H.Crane	Ribbon-like	epiphytic/epilithic	Chen et al. (2013)
26	Pteridaceae	*Haplopteris ensiformis* (Sw.) E.H.Crane	Ribbon-like	epiphytic/epilithic	Chen et al. (2013)
27	Pteridaceae	*Haplopteris flexuosa* (Fée) E.H.Crane	Ribbon-like	epiphytic/epilithic	Chen et al. (2013)
28	Pteridaceae	*Haplopteris mediosora *(Hayata) X.C.Zhang	Ribbon-like	epiphytic/epilithic	Yoneoka et al. (2023)
29	Pteridaceae	*Haplopteris* sp.	Ribbon-like	epiphytic/epilithic	Kuo et al. (2017)
30	**Aspleniaceae**	***Hymenasplenium murakami-hatanakae Nakaike***	**Strap-like**	**epilithic**	**This study**
31	Lomariopsidaceae	*Lomariopsis kunzeana* (C.Presl) Holttum	Ribbon-like	epilithic	Possley et al. unpublish; Pinson et al. (2017a)
32	Lomariopsidaceae	*Lomariopsis lineata* (C. Presl) Holttum	Ribbon-like	epilithic	Li et al. (2009)
33	Lomariopsidaceae	*Lomariopsis* sp.	Strap-like	epiphytic/epilithic*	Ebihara et al. (2013)
34	Polypodiaceae	*Moranopteris nimbata* (Jenman) R.Y.Hirai & J.Prado	Strap-like	epiphytic/epilithic	Farrar (1967)
35	Polypodiaceae	*Loxogramme grammitoides* (Baker) C.Chr	Ribbon-like	epiphytic/epilithic	Ebihara et al. (2013); Park et al. (2020)
36	Polypodiaceae	*Pleurosoriopsis makinoi* (Maxim. ex Makino) Fomin	Ribbon-like	epilithic	Ebihara et al. (2013); Park et al. (2020)

On the other hand, the morphological and functional diversity of fern gametophytes has been well elucidated based on studies of cultivated gametophytes. In describing the diversity of fern gametophyte morphology, Nayar and Kaur (1971) identified five morphological types of fern gametophytes: tuberous, filamentous, cordate thalloid, strap-like, and ribbon-like. Imaichi (2013) proposed another classification system based on developmental types, distinguished by their detailed observations of the cell lineages of gametophytes, and recognized five types: *Lygodium*, *Elaphoglossum*, *Anemia*, *Colysi*s, and *Vittaria*. It was also clarified that the morphological and developmental types are similar within each fern lineage.

Morphology of fern gametophytes was shown to be related to their ecology. According to previous studies, cordate gametophytes are found in the majority of ferns and are characterized by rapid growth and short lifespan, whereas non-cordate gametophytes are found in limited epiphytic or epilithic fern taxa and are characterized by slow growth and long lifespan (Nayar and Kaur 1971; Watkins et al. 2007). Recently, Nitta et al. (2020) conducted a comprehensive correlation analysis of the relationship between gametophyte morphology and their habitats using gametophytes collected in the field. The results revealed a tendency for gametophyte morphology to converge toward a non-cordate form in epiphytic or epilithic ferns. These previous studies demonstrated that the morphological diversity of gametophytes is related to sporophytic habitats.

Moreover, there is a close relationship between the morphological types of gametophytes and the ability to form independent gametophytes. The previous studies showed that the independent gametophytes are observed only in the taxa producing non-cordate gametophytes, for example, in *Vandenboschia* and *Crepidomanes* with filamentous; in *Hymenophyllum*, *Vittaria*, *Haplopteris*, *Antrophyum*, *Loxogramme*, and *Pleurosoriopsis* with ribbon-like; and in *Moranopteris* and *Lomariopsis* with strap-like gametophytes (Table 1). In contrast, no independent gametophytes have been reported in the taxa that produce cordate gametophytes. This unbalanced distribution of independent gametophytes in limited fern groups may be due to the predominance of terrestrial ferns in the major groups of ferns producing cordate gametophytes. In other words, their terrestrial habitats are constantly exposed to disturbances, such as lots of fallen leaves and others, and it is too hard for independent gametophytes to survive, which need long-term stable habitats (Watkins et al. 2007). This may be the reason why the morphology of gametophytes and the ability to be independent gametophytes are closely related.

So far, no independent gametophytes have been reported from the suborder Aspleniineae (eupolypod II) sense PPG1, one of the largest lineages in ferns, which usually form cordate gametophytes. Based on the morphological observations of the cultivated gametophytes, it has been reported that the majority of taxa in this lineage form cordate gametophytes that grow in terrestrial habitat (Momose 1967; Nayar and Kaur 1971; Nitta et al. 2020). However, elongated cordate (strap-like) gametophytes have been observed in certain taxa, particularly epiphytic and epilithic taxa. For example, such gametophytes have been reported in epilithic *Asplenium wrightii* D.C.Eaton ex Hook., *A. tenuicaule* Hayata (Yasuda 2018), *Hymenasplenium hondoense* (N. Murak. & S.-I. Hatan.) Nakaike (Momose 1967) in Aspleniaceae. In addition, strap-like gametophytes have recently been recorded based on wild gametophytes of hemi-epiphytic *Hymenasplenium volubile* (N.Murak. & R.C.Moran) L.Regalado & Prada (Watts et al. 2019). However, these strap-like gametophytes, thought to be recently derived from cordate gametophytes, have not been reported to be capable of vegetative reproduction. Therefore, fern independent gametophytes capable of maintaining their populations for long periods of time have not yet been recorded from the Aspleniineae.

**Fig. 1:**
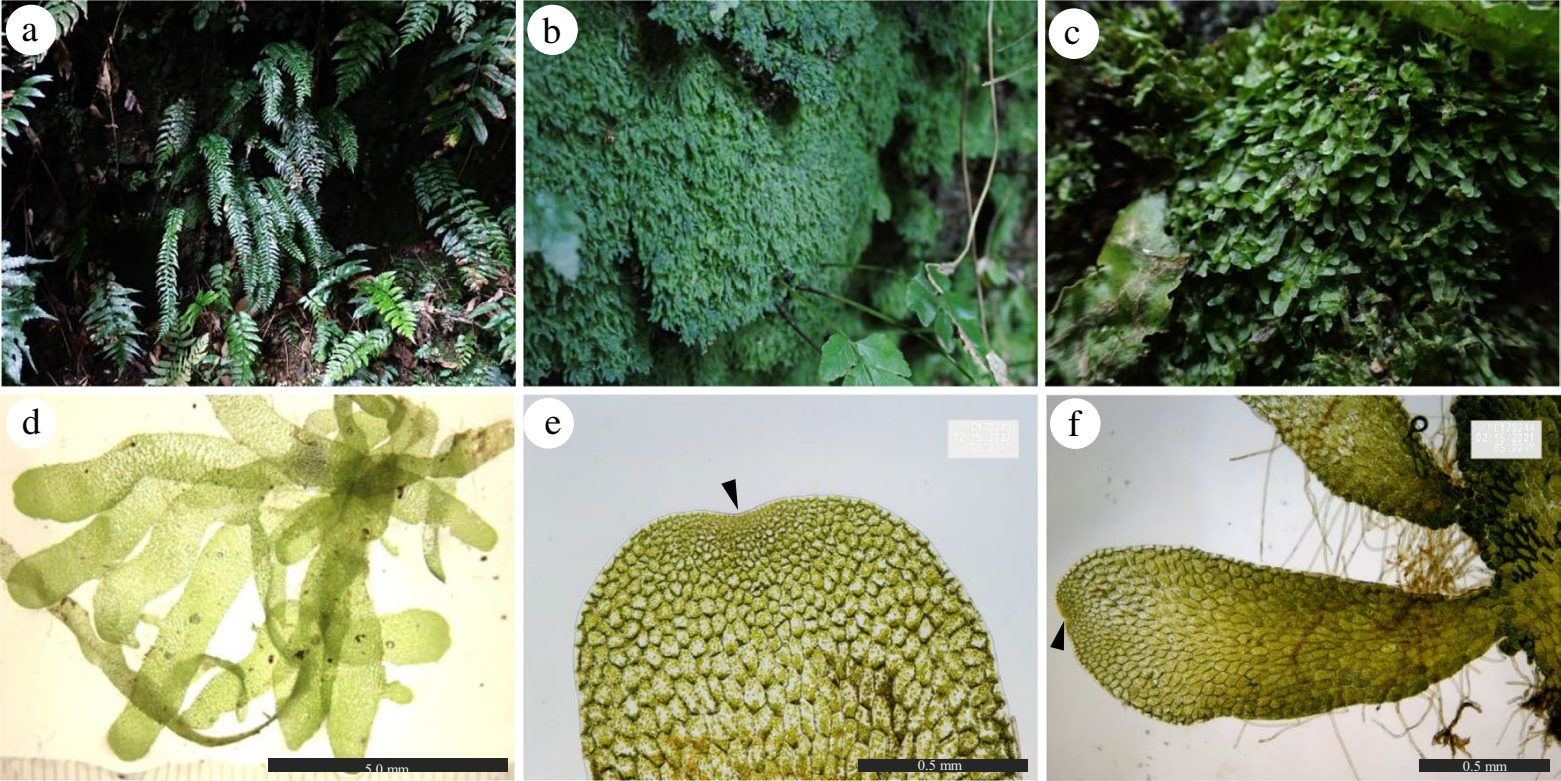
The sporophytes and gametophytes of *Hymenasplenium murakami-hatanakae*. **a** The sporophytes found in Nanami-sawa, Izu-Oshima Island, Tokyo Pref., Japan.; **b, c** The gametophyte mats discovered in Shikamaga-taki, Izu-Oshima Island, Tokyo Pref., Japan.; **d** The gametophyte individuals observed under a microscope. The scale bar indicates 5.0 mm., **e** The top of thallus. Black arrow indicates the position of multiple meristems. The scale bar, 0.5 mm.; **f** The clonal prothalli. Black arrow indicates the multiple meristems of prothallus

By chance, we found many strap-like gametophyte mats, presumed to be those of *Hymenasplenium murakami-hatanakae* Nakaike, near the conspecific sporophyte populations on Izu-Oshima Island, the Izu Islands, Tokyo Pref., Japan (Fig. 1). This fern species belongs to the family Aspleniaceae and is widely distributed in the warm temperate regions from Japan to Taiwan (Ebihara 2016; Murakami and Hatanaka 1988). Izu-Oshima is the locality at the northern limit of its distribution, which we found very recently (Yoneoka et al. 2024). The sporophytes grow on the moist rocky cliffs in deep shade along the streams. In contrast, the gametophytic morphology and ecology of this species have never been reported. In the vicinity of the gametophyte populations, sporophytes of fern species forming strap-like gametophytes, such as those of *Moranopteris* and/or *Lomariopsis*, were not observed. Therefore, the gametophytes were estimated to be those of *H. murakami-hatanakae*.

In this study, we hypothesized that *H. murakami-hatanakae* can form independent gametophytes. We aim to answer the following three questions by careful morphological observation, comparison of chloroplast DNA haplotypes with those of its sporophytes, and comparison of the relative growth positions of gametophytes and sporophytes of *H. murakami-hatanakae* in the natural habitats on Izu-Oshima Island, (1) Can the gametophytes of *H. murakami-hatanakae* produce clonal prothalli such as gemma and reproduce asexually through them? (2) Can this species maintain its gametophyte populations without new spore supply from nearby growing conspecific sporophytes? (3) How extensive is the habitat of the growing gametophytes compared to its conspecific sporophytes?

## Materials and methods

### Field survey

The sporophytes of *Hymenasplenium murakami-hatanakae* and their gametophytes were comprehensively searched and collected on the five islands in the Izu Islands, Japan: Izu-Oshima, Miyakejima, Mikurajima, Hachijojima, and Aogashima Islands, from July 2020 to August 2022. The collected sporophytes were identified based on the description given by Murakami and Hatanaka (1988). To avoid collecting clonal individuals produced by the long-creeping rhizomes, the samples were collected paying attention to the growing direction of the rhizomes. The gametophyte samples were collected by patches according to Ebihara et al. (2013) at intervals of at least 1.0 m. To effectively collect the gametophytes of this species, the deeply shaded habitats along small streams, which sporophytes of the fern species prefer, were selectively searched in all the islands examined. In each locality of sample collection, GPS data, altitude, vegetation around the collection sites, and the size of gametophyte mats was recorded. Voucher specimens of all the samples were deposited as vouchers in the Makino Herbarium of Tokyo Metropolitan University (MAK).

To clarify relative growing positions of sporophytes and gametophytes of *H. murakami-hatanakae* on Izu-Oshima Island, two transects of 50 m × 4 m (horizontal × vertical) were established at Nanami-sawa and Shikamaga-taki waterfalls along the rock wall. These transects were subdivided into 1 m × 4 m sections, and presence or absence of sporophytes and/or gametophyte mats over 5 cm in diameter was recorded in each section. Species identification of sporophytes and gametophytes was done based on the nucleotide sequences of the chloroplast *rbcL* gene, as will be explained in detail in the following section.

To explore the potential statistical association between sporophyte and conspecific gametophyte distributions, Fisher's exact test using presence-absence data was conducted. This test was chosen because it is suitable for analyzing independence or association in categorical variables, particularly when sample sizes are small or expected counts are low. The null hypothesis tested was no significant association between sporophyte and conspecific gametophyte distributions. A 2 × 2 contingency table illustrated joint occurrences observed, with expected counts calculated by assuming independence. Fisher's Exact Test statistic was computed, and the resulting p-value was compared to a predetermined significance level (e.g., 0.05) to infer the rejection or acceptance of the null hypothesis. All analyses were performed using R version 4.2.2.

### DNA barcoding of the gametophytes and cpDNA haplotype analysis

The total DNA was extracted from each sporophyte and gametophyte sample (about 0.5 cm × 0.5 cm part) using the CTAB methods of Doyle and Doyle (1987). Chloroplast *rbcL* gene (*rbcL*) and the *trnL-F* intergenic spacer sequences including *trnL* intron (*trnL-L-F*) were amplified selectively in polymerase chain reaction (PCR), with Prime STAR Max DNA Polymerase (Takara, Kyoto, Japan) under the following conditions: 95 °C for 7 min, followed by 35 cycles of 98 °C for 10 s, 58 °C for 15 s, and 72 °C for 8 s, and 72 °C for 7 min. The sequences of the PCR primers, which were used in this study, are shown in Table 2. Raw PCR products were purified using the Exo SAP-IT Express PCR Product Cleanup Reagent (USB Products Affymetrix, Inc., Cleveland, USA) and used as templates for direct sequencing. Reaction mixtures for sequencing were prepared using the Super Dye Cycle Sequencing Kit v3.1 (Edge BioSystems, California, USA). The reaction mixtures were analyzed using an ABI 3130 Genetic Analyzer (Applied Biosystems, California, USA).

**Table 2:** Primers used in this study. Target region, primer name, primer sequence and reference are given for each primer

Target region	Primer name	Primer sequence	Reference
*rbcL*	af3	5’- ATGTCACCACAAACGGAGACTAAAGC-3’	Hori et al. (2018)
*rbcL*	cr3	5’- GCGGCAGCCAATTCCGGACTCCA-3’	Hori et al. (2018)
*trnL-L-F*	FernL 1Ir1	5’- GGYAATCCTGAGCCAAATC -3’	Li et al. (2009)
*trnL-L-F*	F	5’- ATTTGAACTGGTGACACGAG -3’	Taberlet et al. (1991)

For DNA barcoding, *rbcL* was selected because these markers were frequently used for phylogenetic study of the genus *Hymenasplenium* and the taxon coverage is higher than the other chloroplast DNA regions. In total, 29 sequences, comprising 27 taxa of the *Hymenasplenium* and two species of *Asplenium* as outgroups were downloaded from GenBank (Table S1). After the newly obtained sequences in this study were incorporated into the dataset, the sequence matrix was aligned using MUSCLE (Edgar 2004) followed by manually checking in Aliview (Larsson 2014). SYM + I + G was adopted as the best substitution model for *rbcL* based on AIC (Akaike 1974) using jModelTest 2.1.10 (Darriba et al. 2012). Subsequently, the phylogenetic tree was reconstructed using three different methods: bayesian inference (BI), maximum likelihood (ML), and maximum parsimony (MP). BI analyses were conducted using MrBayes 3.2.6 (Ronquist et al. 2012), two runs of four MCMC chains for 100,000,000 generations with samples taken every 1,000 generations were employed. Tracer 1.6 (Rambaut et al. 2018) was used to evaluate the sampled trees with a focus on convergence and effective sample size (ESS). The first 25.0% of sample trees from each run were discarded as burn-in periods. ML analyses were performed using IQ-TREE v.1.6 (Nguyen et al. 2015), the general bootstrap method with 1,000 replications was employed to estimate the confidence levels of monophyletic groups. MP analysis was performed using a heuristic approach with TBR branch swapping, as implemented in MEGA-X (Kumar et al. 2018). Ten initial trees were generated by the addition of randomly selected sequences. The robustness of each branch was assessed by bootstrap analysis based on 1,000 replicates. Each gametophyte was identified as *H. murakami-hatanakae* based those obtained from the conspecific sporophyte which was collected in Yoneoka et al. (2024).

To investigate the independence of gametophyte populations from the conspecific sporophyte, cpDNA haplotype analyses were conducted. All the collected sporophytes and the gametophyte mats of *H. murakami-hatanakae* were used for this comparison. The cpDNA haplotypes were identified based on Single nucleotide polymorphisms (SNPs) and insertion/deletion (indel) within *trnL*-*L-F*.

### Morphological observations of gametophytes

For morphological observations of the gametophytes, which were identified as *H. murakami-hatanakae* based on the obtained nucleotide sequence data, they were washed with distilled water to remove bryophytes and soils under a stereo microscope LEICA EZ4 HD. To classify the development types of gametophytes and to clarify the possibility of vegetative reproduction, we recorded the size of gametophytes, the position of meristems, the shape of wings, the thickness of the cushion, the number of gemma-like structures (prothalli) per individual, and the size of prothalli under an optical microscope (Leica DM 2500, Leica Microsystems, Heerbrugg, Switzerland). Additionally, we observed the presence or absence of sexual organs, the size of cells on the marginal and central parts of the gametophytes, the depth of the notch, and the shape of rhizoids. In this study, the development types of gametophytes were classified based on the general definition of Nayar and Kaur (1971), in addition to the new definition of Imaichi (2013), which was suggested based on her observation of detailed cell lineages.

## Results

### The sporophytes and gametophytes of Hymenasplenium murakami-hatanakae

In total, 26 individuals of *Hymenasplenium* sporophytes were collected from three different sites: Nanami-sawa in Izu-Oshima, Mt. Higashiyama in Hachijojima, and Yasundo-gou in Aogashima. These sporophytes exhibit a long-creeping rhizome, once-pinnate laminae with sickle-shaped pinnae, and sorus formation on the margin of pinnae. Additionally, their *rbcL* sequences were completely identical to those of the sporophytes of *H. murakami-hatanakae*, previously identified by Yoneoka et al. (2024) for fern species identification. Therefore, these sporophytes were identified as *H. murakami-hatanakae* based both on morphological characteristics and DNA barcoding. In our observations of sporophytes in the Izu Islands, Hachijojima Island had the largest populations across the island, while sporophytic habitats on Aogashima Island and Izu-Oshima Island were more restricted. Each population was found on the rocky cliff near a stream or in the old explosion crater formed in the late Pleistocene under the evergreen broad-leaved forests. The microhabitats were constantly damp and poorly lit. Despite careful surveys based on voucher information, the sporophytes could not be found on Miyakejima and Mikurajima Islands.

**Fig. 2:**
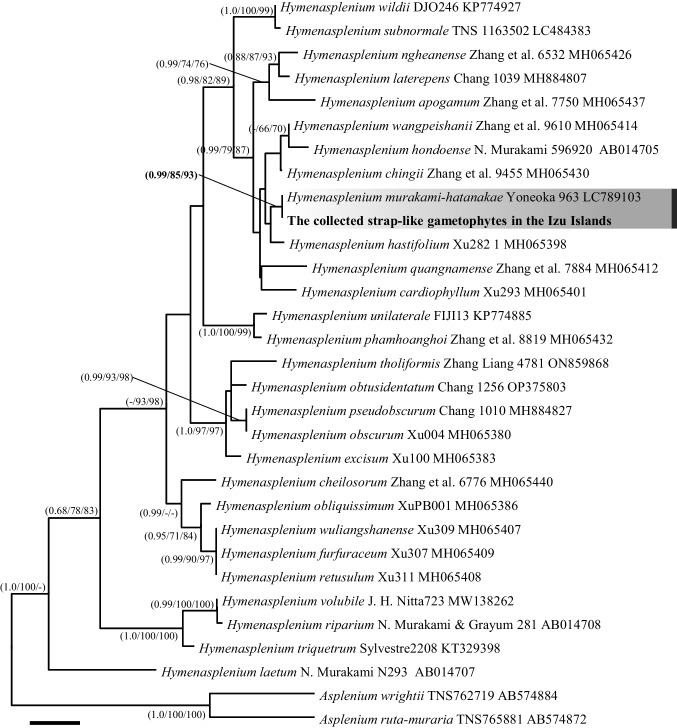
The maximum parsimony tree of *Hymenasplenium* based on *rbcL* sequences. Support values are shown as PP/BS/MP above the branches. The *H. murakami-hatanakae* clade is highlighted by gray squares. The sequence of collected gametophytes collected in the Izu Islands is shown in bold. Scale bar, 10, number of nucleotide substitutions

**Fig. 3:**
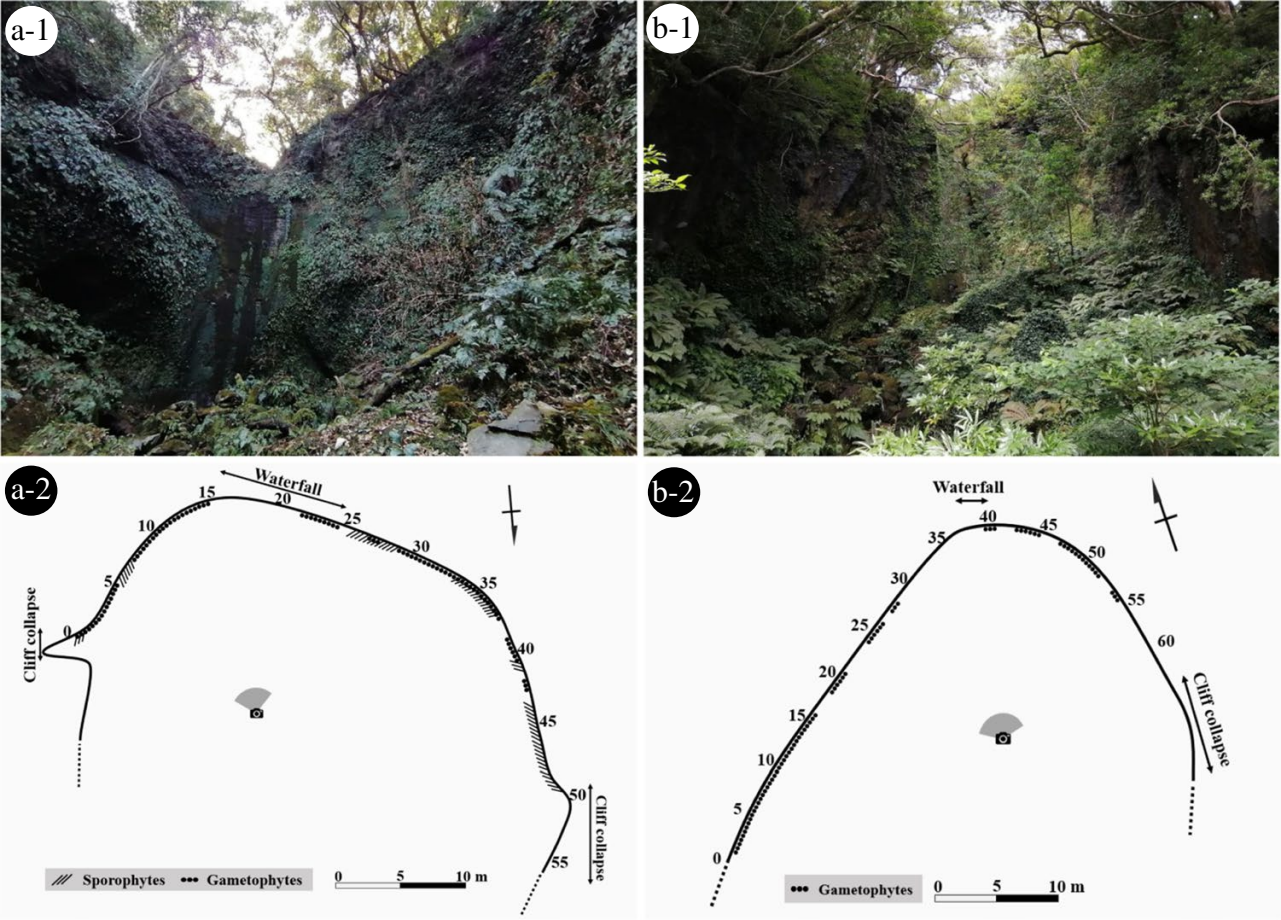
Two collection sites in Izu-Oshima Island **a-1** Nanami-sawa, Senzu **b-1** Shikamaga-taki, Sashikiji **a-2**, **b-2** Comparison of the relative position between the sporophytes of *Hymenasplenium murakami-hatanakae* and conspecific gametophytes in each site. Black diagonal lines, sporophyte. Black dots, gametophytes. The scale bar, 10 m. The black arrow indicates the direction of north

A total of 105 gametophytes, identified as *H. murakami-hatanakae* (Fig. 2), were found at four collection sites: Shikamaga-taki on Izu-Oshima Island, 10.42 km south of Nanami-sawa, and around three sporophyte populations. Despite careful field surveys, we could not find the gametophytes on Miyakejima and Mikurajima Islands. The discovered gametophytes existed as mats basically on the moist rocky cliff, and some of them expanded to the boulders, the slope of soil, and the artificial structures such as a stone monument or concrete wall (Fig. 1). The size of mats was usually from 5 to 30 cm in diameter, while the largest one reached approximately 8 m × 4 m (horizontal × vertical). Most notably, the gametophyte mats were observed even under the lighter environments, where their sporophytes are unable to grow. For example, due to the undeveloped and open canopy of the forest, strong sunlight reached to the surface of a rock cliff at the Shikamaga-taki. Around this site, there was no suitable environment for the sporophytes of *H. murakami-hatanakae* within at least a 10 km radius, while at least 41 gametophyte mats grew without conspecific sporophytes on the surface of the rocky cliff for a width of 50 m (Fig. 3).

The results of our investigation of the presence or absence of sporophytes and conspecific gametophyte mats along the transect at Nanami-sawa are shown in Fig. 3 and Table S3. The following results were obtained using Fisher's exact test: the obtained p-value was 0.02599. The systematic sampling provided detailed data on the distribution patterns of the two generations of *H. murakami-hatanakae*. Specifically, among the sampled sections, 5 sections showed the coexistence of both sporophytes and gametophytes, 10 sections showed the presence of only sporophytes, 24 sections showed the presence of only gametophytes, and 10 sections showed the absence of both sporophytes and gametophytes. The sections where sporophytes and gametophytes coexisted sympatrically were the fewest and most restricted compared to sections under other conditions. These detailed counts contributed to the calculation of the Fisher's Exact Test statistic. The obtained p-value is below the predetermined significance level of 0.05, leading to the rejection of the null hypothesis, indicating a statistically significant association between the presence or absence of sporophytes and conspecific gametophytes. In other words, the presence and absence pattern of two generations of *H. murakami-hatanakae* in Nanami-sawa was not caused by chance.

### Morphological observations of the gametophytes

The gametophytes of *H. murakami-hatanakae* exhibited the following morphological characteristics (Fig. 1d): the mean size of the gametophytes was 11.40 mm × 1.17 mm (length × width). The shallow notch (multicellular meristem) existed at the top of thallus (Fig. 1e). The wings formed a generally symmetrical shape on both sides (Fig. 1e). Therefore, the morphotype is strap-like based on Nayar and Kaur (1971), and the developmental type is classified as Elaphoglossum-type based on Imaichi (2013). The central cushion was underdeveloped and, archegonia and antheridia were absent. Linear rhizoids were produced along both sides of the thallus margin. The average cell size and shape differed between the central and marginal part of the gametophyte. In the central part, the cells were elongated, measuring 143.4 µm × 58.5 µm, in contrast they exhibited a more square-like shape, measuring 81.8 µm × 63.2 µm.

As a result of observations focused on the ability of vegetative reproduction, gemmae-like structures (clonal prothalli) produced through clonal forming were observed on most gametophyte individuals (Fig. 1e). The clonal prothalli were attached to the edges of the thallus, ranging from 0 to 3. The attached positions were away from the multiple meristems of the thallus. The size of the prothalli ranged from 0.6 mm × 0.15 mm (length × width) to 4.2 mm × 0.8 mm. A shallow notch was present at the apex of each prothallus (Fig. 1f). Rhizoids were slightly produced on both sides of the margins of the prothalli. In several lineages of ferns that form independent gametophytes, the gametophyte populations are known to be maintained by branching, but such branching was not observed in the gametophytes of *Hymenasplenium murakami-hatanakae*. The gametophyte mats are maintained solely by vegetative reproduction of prothalli.

### The comparison of cpDNA haplotype frequencies between the sporophyte and gametophyte populations

The chloroplast DNA haplotype analyses demonstrated 3 haplotypes from the Izu Islands (Table 3). Each haplotype was identified based on several insertion to the *trnL-L-F* sequence. All the haplotypes were found from the sporophyte generation, and those of 2 haplotypes were obtained from the gametophyte generation. In the sporophytes, the frequency was highest for haplotype A (88.46%), followed by haplotype B (7.69%) and haplotype C (3.85%). Whereas in the conspecific gametophytes, haplotype A (60.00%) and haplotype B (40.00%) were observed (Table S2).

**Table 3:** Insertions in *trnL-L-F* haplotypes of *Hymenasplenium murakami-hatanakae* in the Izu Islands

cp DNA haplotypes	Accession #	Site																																				
		11									20										30										40						46	185
Haplotype A	**LC815043**	-	-	-	-	-	-	-	-	-	-	-	-	-	-	-	-	-	-	-	-	-	-	-	-	-	-	-	-	-	-	-	-	-	-	-	-	-
Haplotype B	**LC815042**	T	T	G	A	A	T	A	A	A	T	T	C	A	A	A	C	G	A	-	-	-	-	-	-	-	-	-	-	-	-	-	-	-	-	-	-	-
Haplotype C	**LC815041**	T	T	G	A	A	T	A	A	A	T	T	C	A	A	A	C	G	A	T	T	G	A	A	T	A	A	A	T	T	C	A	A	A	C	G	A	G

**Fig. 4:**
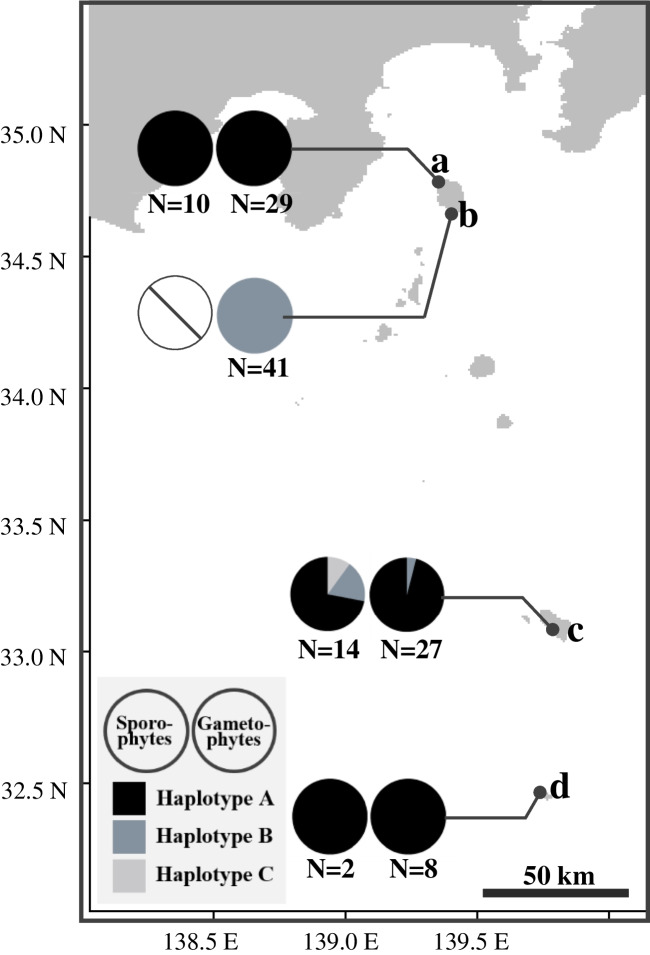
Locations of the sporophyte and gametophyte populations of *Hymenasplenium murakami-hatanakae* examined in this study. The left pie chart shows the haplotype frequencies of cp DNA in the sporophyte generation, while the right pie chart shows those in the gametophytes. The scale bar indicates 50 km. **a** Nanami-sawa, Izu-Oshima Island; **b** Shikamaga-taki, Izu-Oshima Island; **c** Mt. Higashiyama, Hachijojima Island; **d** Yasundo-gou, Aogashima Island. All islands are part of the Izu Islands, Tokyo Pref., Japan

The cpDNA haplotype diversity was highest in the Mt. Higashiyama population on Hachijojima Island, where all three haplotypes were present in the sporophytes, while haplotypes A and B were observed in the gametophytes. In contrast, other populations were fixed for a single cpDNA haplotype, with no haplotypic diversity within each population. For example, the Yasundo-gou population on Aogashima Island and the Nanami-sawa population on Izu Oshima Island consisted of sporophytes and gametophytes with haplotype A. In the Shikamaga-taki population on Izu Oshima Island, only gametophytes of haplotype B were observed without the conspecific sporophytes (Fig. 4).

## Discussion

### Discovery of the strap-like gametophytes of Hymenasplenium murakami-hatanakae with clonal forming

The strap-like gametophytes discovered at Nanami-sawa on Izu-Oshima Island were confirmed through DNA barcoding to belong to *Hymenasplenium murakami-hatanakae*. The phylogenetic tree, constructed using nucleotide sequences of the *rbcL* gene, exhibited a perfect match between the sequences of the obtained strap-like gametophytes and the sporophytes of *H. murakami-hatanakae* (1,205/1,205 bp), forming a single clade with high support bootstrap values, while these sequences were distinct from all other *Hymenasplenium* species included in the phylogenetic analysis (Fig. 2). The *rbcL* gene is commonly utilized for molecular species identification in ferns (Ebihara et al. 2010; Yatabe et al. 2009). Particularly within the genus *Hymenasplenium*, sufficient polymorphism has been demonstrated in the sequences, enabling discrimination among all recognized species to date (Murakami 1995; Xu et al. 2018). Therefore, the identification of these strap-like gametophytes as *H. murakami-hatanakae* is confidently supported by molecular evidence.

Morphological observations using wild gametophytes of *H. murakami-hatanakae* demonstrated their ability to form independent gametophytes through vegetative reproduction. Observations under a microscope revealed gemma-like structures at the periphery of the gametophytes (Fig. 1f). These prothalli originate from the differentiation of marginal body cells of the gametophyte to form a new meristem. The prothalli grow by connecting to the mother gametophytes as clonal individuals. Due to the absence of clonal prothalli larger than 4.2 mm, it is estimated that they eventually separate from their mother gametophytes and either become part of the gametophyte mat or disperse through water flow to distant places. Such gametophytes, possessing the ability of vegetative reproduction, are commonly observed in independent gametophytes of ferns (Farrar 1967; Pinson et al. 2017a). In the genus *Hymenasplenium*, some species are known to form strap-like gametophytes (Momose 1967; Watts et al. 2019), but it has been believed that these gametophytes lack the ability of vegetative reproduction, or the functional significance of the peculiarly shaped gametophytes has not been emphasized. This study represents the first description of the capability of strap-like gametophytes of *Hymenasplenium* to sustain their gametophytic populations through vegetative reproduction via clonal forming, based on detailed morphological and field observations.

Furthermore, independent gametophyte populations were found on two other islands in the Izu Islands, namely Aogashima and Hachijojima, as well as another locality on Izu-Oshima: Shikamaga-taki (Fig. 4). To explore potentially suitable habitats for *H. murakami-hatanakae*, surveys of the gametophyte mats were conducted along moist rocky areas adjacent to shaded streams, where sporophytes of the fern species could potentially grow, and the strap-like gametophyte mats, estimated as those of this fern species, were selectively collected. Based on DNA barcoding using *rbcL*, all the gametophyte mats collected near the sporophyte population on Hachijojima Island and Aogashima Island were identified as those of *H. murakami-hatanakae*. Remarkably, the gametophytes found at Shikamaga-taki on Izu-Oshima Island without their conspecific sporophytes were also identified as *H. murakami-hatanakae*. It was also elucidated that these gametophytes possess the ability of clonal forming for vegetative reproduction. The discovery of these gametophyte mats, sustained by clonal forming across a wide area in the Izu Islands, suggests that *H. murakami-hatanakae* may have maintained the gametophyte population for a long period of time by vegetative reproduction.

### The independent gametophytes of Hymenasplenium murakami-hatanakae

This study clarified that *H. murakami-hatanakae* can indeed form independent gametophytes, as evidenced by the results of cpDNA haplotype analysis. The investigation of 26 sporophytes and 105 gametophytes collected from four locations in the Izu Islands resulted in the identification of three cpDNA haplotypes distinguished by indels (Table 3). Initially, the gametophyte population without conspecific sporophytes in the vicinity (within a 10 km radius) of Shikamaga-taki on Izu-Oshima Island was expected to receive spore supply from mature sporophytes growing in Nanami-sawa in the northern part of the same island. However, these two populations had completely different cpDNA haplotypes. The haplotype B observed in the gametophyte population at Shikamaga-taki was not found in those at Nanami-sawa in the same Izu-Oshima, and was a rare haplotype found only in a few sporophytes on Hachijojima Island (Fig. 4). In ferns, cpDNA in the gametophyte is directly inherited from the mother sporophyte (Raghavan 1980). In other words, the differences in cpDNA haplotypes can be attributed to differences in the spore parents (Pinson and Schuettpelz 2016; Yoneoka et al. 2023). In the case of Izu-Oshima, the origin of the gametophyte population at Shikamaga-taki is likely from outside of the island, not from the sporophyte populations at Nanami-sawa on the same island. Furthermore, as demonstrated later, the very large Shikamaga-taki gametophyte population has likely persisted for long periods, suggesting its origin from long-distance spore dispersal and subsequent vegetative reproduction.

More precisely, according to the definition provided by Kuo et al. (2017), we could recognize the presence of two distinct levels of independence within the identified gametophyte populations in the Izu Islands: facultative and obligate independence. In the survey of independent gametophytes in the Izu Islands, we discovered 105 gametophyte mats. Among them, we recognized facultative instances in Nanami-sawa on Izu-Oshima, and in several populations on Hachijojima and Aogashima Islands (Fig. 4). In these populations, abundant gametophyte mats are found adjacent to the mature sporophytes of *H. murakami-hatanakae*. These gametophyte mats covered moist cliff surfaces, surpassing the extent of sporophyte growth. Genetic analysis of the collected gametophytes indicated that, at each site, sporophytes with the same cpDNA haplotype as those of gametophytes coexisted near the gametophyte mats (Fig. 4). These gametophyte populations are estimated as facultative independent gametophytes, receiving frequent spore supply from nearby conspecific sporophytes and sustaining themselves through vegetative reproduction. In contrast, we recognized obligate instances at Shikamaga-taki on Izu-Oshima Island. In this locality, the gametophyte population has been maintained without conspecific sporophytes. Although the sporophytes of *H. murakami-hatanakae* were found in a limited area at Nanami-sawa in the northern part of Izu-Oshima, most of the island is estimated to be outside the geographical range for sporophyte growth (Yoneoka et al. 2024), primarily because this island is too open, dry, and cold for the fern species. The large gametophyte mat extending over 50 m in Shikamaga-taki suggests the sustained existence of the gametophyte population since its establishment for a very long period more than several hundred years (Fig. 3). These results collectively support the classification of the Shikamaga-taki population as obligate independent gametophytes.

### Ecological habitat differentiation between the sporophyte and gametophyte

*Hymenasplenium murakami-hatanakae* showed notable differences in growing habitats between the sporophytes and gametophytes. During our first survey in Nanami-sawa on Izu Oshima Island, we noticed that sporophytes and gametophyte mats may grow separately on the rock walls. To precisely show the difference in habitats between the two generations, a systematic sampling approach was implemented on Izu Oshima. The investigation of Nanami-sawa revealed that the gametophytes grew in a broader range of areas than the sporophytes, as shown in Fig. 3. Statistical analysis, using the Fisher's exact test, provided support for the presence of statistical heterogeneity in the data concerning the presence or absence of sporophytes and gametophytes (*p* < 0.05). Notably, the test results indicated a potential disparity in the habitats where each generation grows, given the higher number of areas where only gametophytes or only sporophytes grow compared to the areas where both coexist.

The environmental factors most likely associated with the independent gametophyte formation of *H. murakami-hatanakae* were shade of sunlight and humidity. To estimate the environmental factors for the clear differentiation of growing habitats between the two generations, special attention was given to the environmental differences between the two populations within Izu-Oshima, specifically at Shikamaga-taki and Nanami-sawa. Shikamaga-taki, where only gametophytes of this fern species grow, had only half-shaded, south-facing rock walls, providing relatively dry conditions throughout the year except just after heavy rainfall (Fig. 3). In contrast, at Nanami-sawa, where sporophytes are present, was in deep shade without direct sunlight due to the presence of wet, rocky areas that retain water throughout the year and a north-facing slope that is completely covered by a canopy of evergreen forest (Yoneoka et al. 2024). Notably, within Izu-Oshima, an island where both sporophytes and gametophytes are observed, the significant environmental differences between the sites with and without visible sporophytes are intriguing for estimating the environmental factors maintaining the gametophyte mats. Previous studies have reported variations in environmental tolerance between sporophytes and gametophytes of ferns (Ebihara et al. 2019; Farrar 1998; Makgomol and Sheffield 2001; Nitta et al. 2021; Sato and Sakai 1981; Watkins et al. 2007). Such physiological and ecological differences have been discussed as key factors causing differences in distribution and niche preferences between the two generations. The distinct spatial separation of the gametophytes and sporophytes of *H. murakami-hatanakae* suggests differences in ecological preferences between the two generations, such as requirements for light and humidity.

### Evolutionary significance for the discovery of independent gametophytes from Hymenasplenium

*Hymenasplenium murakami-hatanakae* is the first species of Aspleniineae (eupolypod II) of which fern independent gametophytes were found. This study clearly demonstrates that the gametophytes of this species can be sustained by vegetative reproduction through clonal forming without the necessity of new spore supply from sporophytes, as evidenced by clonal forming observations and molecular analysis of cpDNA haplotypes. In previous studies, independent gametophytes have been recognized to be limited to epiphytic families capable of forming non-cordate gametophytes, namely Hymenophyllaceae, Pteridaceae, Lomariopsidaceae, and Polypodiaceae (Farrar 1967; Pinson et al. 2017a). Ferns within the suborder Aspleniineae sensu PPGI, one of the largest lineages in ferns, have been primarily acknowledged as terrestrial species forming typical cordate gametophytes (Nayar and Kaur 1971), and no instances of independent gametophytes have been reported from this suborder (Table 1). The discovery of a species belonging to Aspleniineae that can undergo a significant transformation in the lifestyle of gametophytes to form independent gametophytes is the most noteworthy achievement of this study.

The diversification of gametophyte morphology and the ability to form independent gametophytes in *H. murakami-hatanakae* may have been acquired during ecological shifts to epilithic habitats. In this study, it was demonstrated that the gametophytes can be strap-like in shape and produce gametophyte mats through continuous vegetative reproduction via clonal forming. The evolutionary and ecological significance of long-lived gametophytes and gametophyte mats with complex three-dimensional morphology has been repeatedly discussed in former studies (Dassler and Farrar 2001; Pitterman et al. 2013; Watkins and Cardelús, 2012). Recently, Nitta et al. (2020) demonstrated that epiphytes, including petrophytes, showed a higher frequency of non-cordate gametophytes. *H. murakami-hatanakae* is also a typical epilithic fern species (Murakami and Hatanaka 1988). The trait of forming long-lived independent gametophytes might be shared with other epilithic or epiphytic species within *Hymenasplenium*, while it is not shared with terrestrial species of the genus, such as *H. excisum* (C.Presl) Hayata and *H. apogamum* (N.Murak.&S.-I. Hatan.) Nakaike. Field observations of gametophytes of other Hymenasplenium species are necessary in future studies.

## Supplementary Information

Below is the link to the electronic supplementary material.

Supplementary file1 (DOCX 45 KB)

## Data Availability

The datasets generated and/or analyzed during the current study are not publicly available due to ownership rights but are available from the corresponding author on reasonable request.
