# Supplementary material for: Morphological and functional evolution of gametophytes in epilithic Hymenasplenium murakami-hatanakae (Aspleniaceae): The fifth family capable of producing the independent gametophytes
Source: J Plant Res. 2024 Jun 25;137(5):815–28. doi: 10.1007/s10265-024-01553-0 (PMC11393290; doi:10.1007/s10265-024-01553-0)
Supplement: Supplementary file 1 — Supplementary file1 (DOCX 45 KB) [file 10265_2024_1553_MOESM1_ESM.docx]

**Supplementary**

**Table S1.** Accession list for phylogenetic analysis.

| Taxon | Voucher specimen | Herbarium Code | Location | *rbcL* |
| --- | --- | --- | --- | --- |
| *Asplenium ruta-muraria* L. | TNS 765881 | TNS | Iwate, Japan | AB574872 |
| *Asplenium wrightii* D.C.Eaton ex Hook. | TNS 762719 | TNS | Miyazaki, Japan | AB574884 |
| *Hymenasplenium apogamum* (N. Murakami & Hatanaka) Nakaike | Zhang et al. 7750 | CDBI, MO, VNMN | Thua Thien-Hue, Vietnam | MH065437 |
| *Hymenasplenium cardiophyllum* (Hance) Nakaike | Xu 293 | SYS | Guangxi, China | MH065401 |
| *Hymenasplenium cheilosorum* (Kunze ex Mettenius) Tagawa | Zhang et al. 6776 | CDBI, MO, VNMN | Bac Kan, Vietnam | MH065440 |
| *Hymenasplenium chingii* K.W. Xu, Li Bing Zhang & W.B.Liao | Zhang et al. 9455 | CDBI | Yunnan, China | MH065430 |
| *Hymenasplenium excisum* (C. Presl) S. Lindsay | Xu 100 | SYS | Hainan, China | MH065383 |
| *Hymenasplenium furfuraceum* (Ching) Viane & S. Y. Dong | Xu 307 | SYS | Yunnan, China | MH065409 |
| *Hymenasplenium hastifolium* Ke Wang Xu, Li Bing Zhang &W. B. Liao | Xu 282-1 | SYS | Guangxi, China | MH065398 |
| *Hymenasplenium hondoense* (N. Murakami & Hatanaka) Nakaike | N. Murakami 596920 | KYO | Kouchi, Japan | AB014705 |
| *Hymenasplenium laetum* (Sw.) L. Regalado & Prada | N. Murakami N293 | KYO | Rio Palenque, Ecuador | AB014707 |
| *Hymenasplenium laterepens* N. Murak. & X. Cheng ex Y.Fen Chang & K. Hori | Chang1039 | HITBC | Xishuangbanna, Yunnan, China | MH884807 |
| *Hymenasplenium murakami-hatanakae* Nakaike | K. Yoneoka 963 (Sporophyte) | MAK | Nanami-sawa, Izu Oshima I., Tokyo, Japan | LC789103 |
| *Hymenasplenium murakami-hatanakae* Nakaike | K. Yoneoka 423 (Gametophyte) | MAK | Shikamaga-taki, Izu Oshima I., Tokyo, Japan | LC815044 |
| *Hymenasplenium ngheanense* Li Bing Zhang, K.W. Xu & N.T.Lu | Zhang et al. 6532 | CDBI, MO, VNMN | Phu Tho, Vietnam | MH065426 |
| *Hymenasplenium obliquissimum* (Hayata) Sugimoto | Xu PB001 | SYS | Yunnan, China | MH065386 |
| *Hymenasplenium obscurum* (Blume) Tagawa | Xu 004 | SYS | Hong Kong, China | MH065380 |
| *Hymenasplenium phamhoanghoi* Li Bing Zhang, K.W. Xu & T.T. Luong | Zhang et al. 8819 | CDBI, MO, PHH | Khánh Hòa, Vietnam | MH065432 |
| *Hymenasplenium pseudobscurum* Viane | Chang1010 | HITBC | Yunnan, China | MH884827 |
| *Hymenasplenium quangnamense* Li Bing Zhang, K.W. Xu & Liang Zhang | Zhang et al. 7884 | CDBI, MO, VNMN | Quảng Nam, Vietnam | MH065412 |
| *Hymenasplenium retusulum* (Ching) Viane & S. Y. Dong | Xu 311 | SYS | Yunnan, China | MH065408 |
| *Hymenasplenium riparium* (Liebm.) L. Regalado & Prada | N. Murakami & Grayum 281 | KYO | Virgen del Socorro, Costa Rica | AB014708 |
| *Hymenasplenium subnormale* (Copel.) Nakaike | TNS 1163502 | TNS | Yonaguni, Okinawa, Japan | LC484383 |
| *Hymenasplenium tholiformis* Liang Zhang, K. W. Xu & W. B. Ju | Zhang Liang 4781 | KUN | Tibet, China | ON859868 |
| *Hymenasplenium triquetrum* (N.Murak. & R.C.Moran) L.Regalado & Prada | Sylvestre 2208 | RB | Brazil | KT329398 |
| *Hymenasplenium unilaterale* (Lam.) Hayata | Brownsey & Perrie FIJI 13 | WELT | Fiji | KP774885 |
| *Hymenasplenium volubile* (N.Murak. & R.C.Moran) L.Regalado & Prada | JH Nitta723 | UC | Nectandra, Costa Rica | MW138262 |
| *Hymenasplenium wangpeishanii* Li Bing Zhang & K.W. Xu | Zhang et al. 9610 | CDBI | Guizhou, China | MH065414 |
| *Hymenasplenium wildii* (F.M.Bailey) D.Ohlsen | Ohlsen 246 | MELU | Queensland, Australia | KP774927 |
| *Hymenasplenium wuliangshanense* (Ching) Viane & S. Y. Dong | Xu 309 | SYS | Yunnan, China | MH065407 |

**Table S2.** List of cpDNA haplotypes.

| **Voucher  specimen** | **Generation** | **cpDNA  Haplotype** | **Location** |
| --- | --- | --- | --- |
| **K. Yoneoka 657** | **Gametophyte** | **A** | **Yasundo-gou, Aogashima Island, the Izu islands, Tokyo Pref., Japan** |
| **K. Yoneoka 658** | **Gametophyte** | **A** | **Yasundo-gou, Aogashima Island, the Izu islands, Tokyo Pref., Japan** |
| **K. Yoneoka 659** | **Gametophyte** | **A** | **Yasundo-gou, Aogashima Island, the Izu islands, Tokyo Pref., Japan** |
| **K. Yoneoka 661** | **Gametophyte** | **A** | **Yasundo-gou, Aogashima Island, the Izu islands, Tokyo Pref., Japan** |
| **K. Yoneoka 662** | **Gametophyte** | **A** | **Yasundo-gou, Aogashima Island, the Izu islands, Tokyo Pref., Japan** |
| **K. Yoneoka 663** | **Gametophyte** | **A** | **Yasundo-gou, Aogashima Island, the Izu islands, Tokyo Pref., Japan** |
| **K. Yoneoka 664** | **Gametophyte** | **A** | **Yasundo-gou, Aogashima Island, the Izu islands, Tokyo Pref., Japan** |
| **K. Yoneoka 665** | **Gametophyte** | **A** | **Yasundo-gou, Aogashima Island, the Izu islands, Tokyo Pref., Japan** |
| **K. Yoneoka 612** | **Gametophyte** | **A** | **Mt. Higashiyama, Hachijojima island, the Izu islands, Tokyo Pref., Japan** |
| **K. Yoneoka 613** | **Gametophyte** | **A** | **Mt. Higashiyama, Hachijojima Island, the Izu islands, Tokyo Pref., Japan** |
| **K. Yoneoka 614** | **Gametophyte** | **A** | **Mt. Higashiyama, Hachijojima Island, the Izu islands, Tokyo Pref., Japan** |
| **K. Yoneoka 615** | **Gametophyte** | **A** | **Mt. Higashiyama, Hachijojima Island, the Izu islands, Tokyo Pref., Japan** |
| **K. Yoneoka 616** | **Gametophyte** | **A** | **Mt. Higashiyama, Hachijojima Island, the Izu islands, Tokyo Pref., Japan** |
| **K. Yoneoka 617** | **Gametophyte** | **A** | **Mt. Higashiyama, Hachijojima Island, the Izu islands, Tokyo Pref., Japan** |
| **K. Yoneoka 618** | **Gametophyte** | **A** | **Mt. Higashiyama, Hachijojima Island, the Izu islands, Tokyo Pref., Japan** |
| **K. Yoneoka 619** | **Gametophyte** | **A** | **Mt. Higashiyama, Hachijojima Island, the Izu islands, Tokyo Pref., Japan** |
| **K. Yoneoka 620** | **Gametophyte** | **A** | **Mt. Higashiyama, Hachijojima Island, the Izu islands, Tokyo Pref., Japan** |
| **K. Yoneoka 621** | **Gametophyte** | **A** | **Mt. Higashiyama, Hachijojima Island, the Izu islands, Tokyo Pref., Japan** |
| **K. Yoneoka 623** | **Gametophyte** | **A** | **Mt. Higashiyama, Hachijojima Island, the Izu islands, Tokyo Pref., Japan** |
| **K. Yoneoka 624** | **Gametophyte** | **A** | **Mt. Higashiyama, Hachijojima Island, the Izu islands, Tokyo Pref., Japan** |
| **K. Yoneoka 626** | **Gametophyte** | **A** | **Mt. Higashiyama, Hachijojima Island, the Izu islands, Tokyo Pref., Japan** |
| **K. Yoneoka 627** | **Gametophyte** | **A** | **Mt. Higashiyama, Hachijojima Island, the Izu islands, Tokyo Pref., Japan** |
| **K. Yoneoka 629** | **Gametophyte** | **A** | **Mt. Higashiyama, Hachijojima Island, the Izu islands, Tokyo Pref., Japan** |
| **K. Yoneoka 631** | **Gametophyte** | **A** | **Mt. Higashiyama, Hachijojima Island, the Izu islands, Tokyo Pref., Japan** |
| **K. Yoneoka 633** | **Gametophyte** | **A** | **Mt. Higashiyama, Hachijojima Island, the Izu islands, Tokyo Pref., Japan** |
| **K. Yoneoka 634** | **Gametophyte** | **A** | **Mt. Higashiyama, Hachijojima Island, the Izu islands, Tokyo Pref., Japan** |
| **K. Yoneoka 635** | **Gametophyte** | **A** | **Mt. Higashiyama, Hachijojima Island, the Izu islands, Tokyo Pref., Japan** |
| **K. Yoneoka 636** | **Gametophyte** | **A** | **Mt. Higashiyama, Hachijojima Island, the Izu islands, Tokyo Pref., Japan** |
| **K. Yoneoka 639** | **Gametophyte** | **A** | **Mt. Higashiyama, Hachijojima Island, the Izu islands, Tokyo Pref., Japan** |
| **K. Yoneoka 640** | **Gametophyte** | **A** | **Mt. Higashiyama, Hachijojima Island, the Izu islands, Tokyo Pref., Japan** |
| **K. Yoneoka 649** | **Gametophyte** | **A** | **Mt. Higashiyama, Hachijojima Island, the Izu islands, Tokyo Pref., Japan** |
| **K. Yoneoka 653** | **Gametophyte** | **A** | **Mt. Higashiyama, Hachijojima Island, the Izu islands, Tokyo Pref., Japan** |
| **K. Yoneoka 654** | **Gametophyte** | **A** | **Mt. Higashiyama, Hachijojima Island, the Izu islands, Tokyo Pref., Japan** |
| **K. Yoneoka 655** | **Gametophyte** | **A** | **Mt. Higashiyama, Hachijojima Island, the Izu islands, Tokyo Pref., Japan** |
| **K. Yoneoka 447** | **Gametophyte** | **A** | **Nanami-sawa, Izu Oshima Island, the Izu islands, Tokyo Pref., Japan** |
| **K. Yoneoka 448** | **Gametophyte** | **A** | **Nanami-sawa, Izu Oshima Island, the Izu islands, Tokyo Pref., Japan** |
| **K. Yoneoka 449** | **Gametophyte** | **A** | **Nanami-sawa, Izu Oshima Island, the Izu islands, Tokyo Pref., Japan** |
| **K. Yoneoka 450** | **Gametophyte** | **A** | **Nanami-sawa, Izu Oshima Island, the Izu islands, Tokyo Pref., Japan** |
| **K. Yoneoka 451** | **Gametophyte** | **A** | **Nanami-sawa, Izu Oshima Island, the Izu islands, Tokyo Pref., Japan** |
| **K. Yoneoka 452** | **Gametophyte** | **A** | **Nanami-sawa, Izu Oshima Island, the Izu islands, Tokyo Pref., Japan** |
| **K. Yoneoka 453** | **Gametophyte** | **A** | **Nanami-sawa, Izu Oshima Island, the Izu islands, Tokyo Pref., Japan** |
| **K. Yoneoka 454** | **Gametophyte** | **A** | **Nanami-sawa, Izu Oshima Island, the Izu islands, Tokyo Pref., Japan** |
| **K. Yoneoka 456** | **Gametophyte** | **A** | **Nanami-sawa, Izu Oshima Island, the Izu islands, Tokyo Pref., Japan** |
| **K. Yoneoka 457** | **Gametophyte** | **A** | **Nanami-sawa, Izu Oshima Island, the Izu islands, Tokyo Pref., Japan** |
| **K. Yoneoka 459** | **Gametophyte** | **A** | **Nanami-sawa, Izu Oshima Island, the Izu islands, Tokyo Pref., Japan** |
| **K. Yoneoka 460** | **Gametophyte** | **A** | **Nanami-sawa, Izu Oshima Island, the Izu islands, Tokyo Pref., Japan** |
| **K. Yoneoka 464** | **Gametophyte** | **A** | **Nanami-sawa, Izu Oshima Island, the Izu islands, Tokyo Pref., Japan** |
| **K. Yoneoka 964** | **Gametophyte** | **A** | **Nanami-sawa, Izu Oshima Island, the Izu islands, Tokyo Pref., Japan** |
| **K. Yoneoka 965** | **Gametophyte** | **A** | **Nanami-sawa, Izu Oshima Island, the Izu islands, Tokyo Pref., Japan** |
| **K. Yoneoka 966** | **Gametophyte** | **A** | **Nanami-sawa, Izu Oshima Island, the Izu islands, Tokyo Pref., Japan** |
| **K. Yoneoka 967** | **Gametophyte** | **A** | **Nanami-sawa, Izu Oshima Island, the Izu islands, Tokyo Pref., Japan** |
| **K. Yoneoka 968** | **Gametophyte** | **A** | **Nanami-sawa, Izu Oshima Island, the Izu islands, Tokyo Pref., Japan** |
| **K. Yoneoka 969** | **Gametophyte** | **A** | **Nanami-sawa, Izu Oshima Island, the Izu islands, Tokyo Pref., Japan** |
| **K. Yoneoka 970** | **Gametophyte** | **A** | **Nanami-sawa, Izu Oshima Island, the Izu islands, Tokyo Pref., Japan** |
| **K. Yoneoka 971** | **Gametophyte** | **A** | **Nanami-sawa, Izu Oshima Island, the Izu islands, Tokyo Pref., Japan** |
| **K. Yoneoka 972** | **Gametophyte** | **A** | **Nanami-sawa, Izu Oshima Island, the Izu islands, Tokyo Pref., Japan** |
| **K. Yoneoka 973** | **Gametophyte** | **A** | **Nanami-sawa, Izu Oshima Island, the Izu islands, Tokyo Pref., Japan** |
| **K. Yoneoka 974** | **Gametophyte** | **A** | **Nanami-sawa, Izu Oshima Island, the Izu islands, Tokyo Pref., Japan** |
| **K. Yoneoka 975** | **Gametophyte** | **A** | **Nanami-sawa, Izu Oshima Island, the Izu islands, Tokyo Pref., Japan** |
| **K. Yoneoka 976** | **Gametophyte** | **A** | **Nanami-sawa, Izu Oshima Island, the Izu islands, Tokyo Pref., Japan** |
| **K. Yoneoka 1285** | **Gametophyte** | **A** | **Nanami-sawa, Izu Oshima Island, the Izu islands, Tokyo Pref., Japan** |
| **K. Yoneoka 1286** | **Gametophyte** | **A** | **Nanami-sawa, Izu Oshima Island, the Izu islands, Tokyo Pref., Japan** |
| **K. Yoneoka 1287** | **Gametophyte** | **A** | **Nanami-sawa, Izu Oshima Island, the Izu islands, Tokyo Pref., Japan** |
| **K. Yoneoka 423** | **Gametophyte** | **B** | **Shikamaga-taki, Izu Oshima Island, the Izu islands, Tokyo Pref., Japan** |
| **K. Yoneoka 424** | **Gametophyte** | **B** | **Shikamaga-taki, Izu Oshima Island, the Izu islands, Tokyo Pref., Japan** |
| **K. Yoneoka 425** | **Gametophyte** | **B** | **Shikamaga-taki, Izu Oshima Island, the Izu islands, Tokyo Pref., Japan** |
| **K. Yoneoka 428** | **Gametophyte** | **B** | **Shikamaga-taki, Izu Oshima Island, the Izu islands, Tokyo Pref., Japan** |
| **K. Yoneoka 429** | **Gametophyte** | **B** | **Shikamaga-taki, Izu Oshima Island, the Izu islands, Tokyo Pref., Japan** |
| **K. Yoneoka 430** | **Gametophyte** | **B** | **Shikamaga-taki, Izu Oshima Island, the Izu islands, Tokyo Pref., Japan** |
| **K. Yoneoka 431** | **Gametophyte** | **B** | **Shikamaga-taki, Izu Oshima Island, the Izu islands, Tokyo Pref., Japan** |
| **K. Yoneoka 432** | **Gametophyte** | **B** | **Shikamaga-taki, Izu Oshima Island, the Izu islands, Tokyo Pref., Japan** |
| **K. Yoneoka 466** | **Gametophyte** | **B** | **Shikamaga-taki, Izu Oshima Island, the Izu islands, Tokyo Pref., Japan** |
| **K. Yoneoka 467** | **Gametophyte** | **B** | **Shikamaga-taki, Izu Oshima Island, the Izu islands, Tokyo Pref., Japan** |
| **K. Yoneoka 468** | **Gametophyte** | **B** | **Shikamaga-taki, Izu Oshima Island, the Izu islands, Tokyo Pref., Japan** |
| **K. Yoneoka 1253** | **Gametophyte** | **B** | **Shikamaga-taki, Izu Oshima Island, the Izu islands, Tokyo Pref., Japan** |
| **K. Yoneoka 1254** | **Gametophyte** | **B** | **Shikamaga-taki, Izu Oshima Island, the Izu islands, Tokyo Pref., Japan** |
| **K. Yoneoka 1255** | **Gametophyte** | **B** | **Shikamaga-taki, Izu Oshima Island, the Izu islands, Tokyo Pref., Japan** |
| **K. Yoneoka 1256** | **Gametophyte** | **B** | **Shikamaga-taki, Izu Oshima Island, the Izu islands, Tokyo Pref., Japan** |
| **K. Yoneoka 1257** | **Gametophyte** | **B** | **Shikamaga-taki, Izu Oshima Island, the Izu islands, Tokyo Pref., Japan** |
| **K. Yoneoka 1258** | **Gametophyte** | **B** | **Shikamaga-taki, Izu Oshima Island, the Izu islands, Tokyo Pref., Japan** |
| **K. Yoneoka 1259** | **Gametophyte** | **B** | **Shikamaga-taki, Izu Oshima Island, the Izu islands, Tokyo Pref., Japan** |
| **K. Yoneoka 1260** | **Gametophyte** | **B** | **Shikamaga-taki, Izu Oshima Island, the Izu islands, Tokyo Pref., Japan** |
| **K. Yoneoka 1261** | **Gametophyte** | **B** | **Shikamaga-taki, Izu Oshima Island, the Izu islands, Tokyo Pref., Japan** |
| **K. Yoneoka 1262** | **Gametophyte** | **B** | **Shikamaga-taki, Izu Oshima Island, the Izu islands, Tokyo Pref., Japan** |
| **K. Yoneoka 1263** | **Gametophyte** | **B** | **Shikamaga-taki, Izu Oshima Island, the Izu islands, Tokyo Pref., Japan** |
| **K. Yoneoka 1266** | **Gametophyte** | **B** | **Shikamaga-taki, Izu Oshima Island, the Izu islands, Tokyo Pref., Japan** |
| **K. Yoneoka 1267** | **Gametophyte** | **B** | **Shikamaga-taki, Izu Oshima Island, the Izu islands, Tokyo Pref., Japan** |
| **K. Yoneoka 1268** | **Gametophyte** | **B** | **Shikamaga-taki, Izu Oshima Island, the Izu islands, Tokyo Pref., Japan** |
| **K. Yoneoka 1269** | **Gametophyte** | **B** | **Shikamaga-taki, Izu Oshima Island, the Izu islands, Tokyo Pref., Japan** |
| **K. Yoneoka 1270** | **Gametophyte** | **B** | **Shikamaga-taki, Izu Oshima Island, the Izu islands, Tokyo Pref., Japan** |
| **K. Yoneoka 1271** | **Gametophyte** | **B** | **Shikamaga-taki, Izu Oshima Island, the Izu islands, Tokyo Pref., Japan** |
| **K. Yoneoka 1272** | **Gametophyte** | **B** | **Shikamaga-taki, Izu Oshima Island, the Izu islands, Tokyo Pref., Japan** |
| **K. Yoneoka 1273** | **Gametophyte** | **B** | **Shikamaga-taki, Izu Oshima Island, the Izu islands, Tokyo Pref., Japan** |
| **K. Yoneoka 1274** | **Gametophyte** | **B** | **Shikamaga-taki, Izu Oshima Island, the Izu islands, Tokyo Pref., Japan** |
| **K. Yoneoka 1275** | **Gametophyte** | **B** | **Shikamaga-taki, Izu Oshima Island, the Izu islands, Tokyo Pref., Japan** |
| **K. Yoneoka 1276** | **Gametophyte** | **B** | **Shikamaga-taki, Izu Oshima Island, the Izu islands, Tokyo Pref., Japan** |
| **K. Yoneoka 1277** | **Gametophyte** | **B** | **Shikamaga-taki, Izu Oshima Island, the Izu islands, Tokyo Pref., Japan** |
| **K. Yoneoka 1278** | **Gametophyte** | **B** | **Shikamaga-taki, Izu Oshima Island, the Izu islands, Tokyo Pref., Japan** |
| **K. Yoneoka 1279** | **Gametophyte** | **B** | **Shikamaga-taki, Izu Oshima Island, the Izu islands, Tokyo Pref., Japan** |
| **K. Yoneoka 1280** | **Gametophyte** | **B** | **Shikamaga-taki, Izu Oshima Island, the Izu islands, Tokyo Pref., Japan** |
| **K. Yoneoka 1281** | **Gametophyte** | **B** | **Shikamaga-taki, Izu Oshima Island, the Izu islands, Tokyo Pref., Japan** |
| **K. Yoneoka 1282** | **Gametophyte** | **B** | **Shikamaga-taki, Izu Oshima Island, the Izu islands, Tokyo Pref., Japan** |
| **K. Yoneoka 1283** | **Gametophyte** | **B** | **Shikamaga-taki, Izu Oshima Island, the Izu islands, Tokyo Pref., Japan** |
| **K. Yoneoka 1284** | **Gametophyte** | **B** | **Shikamaga-taki, Izu Oshima Island, the Izu islands, Tokyo Pref., Japan** |
| **K. Yoneoka 637** | **Gametophyte** | **B** | **Mt. Higashiyama, Hachijojima Island, the Izu islands, Tokyo Pref., Japan** |
| **K. Yoneoka 977** | **Sporophyte** | **A** | **Yasundo-gou, Aogashima Island, the Izu islands, Tokyo Pref., Japan** |
| **K. Yoneoka 1001** | **Sporophyte** | **A** | **Yasundo-gou, Aogashima Island, the Izu islands, Tokyo Pref., Japan** |
| **K. Yoneoka 980** | **Sporophyte** | **A** | **Mt. Higashiyama, Hachijojima Island, the Izu islands, Tokyo Pref., Japan** |
| **K. Yoneoka 982** | **Sporophyte** | **A** | **Mt. Higashiyama, Hachijojima Island, the Izu islands, Tokyo Pref., Japan** |
| **K. Yoneoka 983** | **Sporophyte** | **A** | **Mt. Higashiyama, Hachijojima Island, the Izu islands, Tokyo Pref., Japan** |
| **K. Yoneoka 986** | **Sporophyte** | **A** | **Mt. Higashiyama, Hachijojima Island, the Izu islands, Tokyo Pref., Japan** |
| **K. Yoneoka 988** | **Sporophyte** | **A** | **Mt. Higashiyama, Hachijojima Island, the Izu islands, Tokyo Pref., Japan** |
| **K. Yoneoka 990** | **Sporophyte** | **A** | **Mt. Higashiyama, Hachijojima Island, the Izu islands, Tokyo Pref., Japan** |
| **K. Yoneoka 991** | **Sporophyte** | **A** | **Mt. Higashiyama, Hachijojima Island, the Izu islands, Tokyo Pref., Japan** |
| **K. Yoneoka 992** | **Sporophyte** | **A** | **Mt. Higashiyama, Hachijojima Island, the Izu islands, Tokyo Pref., Japan** |
| **K. Yoneoka 994** | **Sporophyte** | **A** | **Mt. Higashiyama, Hachijojima Island, the Izu islands, Tokyo Pref., Japan** |
| **K. Yoneoka 995** | **Sporophyte** | **A** | **Mt. Higashiyama, Hachijojima Island, the Izu islands, Tokyo Pref., Japan** |
| **K. Yoneoka 953** | **Sporophyte** | **A** | **Nanami-sawa, Izu Oshima Island, the Izu islands, Tokyo Pref., Japan** |
| **K. Yoneoka 954** | **Sporophyte** | **A** | **Nanami-sawa, Izu Oshima Island, the Izu islands, Tokyo Pref., Japan** |
| **K. Yoneoka 955** | **Sporophyte** | **A** | **Nanami-sawa, Izu Oshima Island, the Izu islands, Tokyo Pref., Japan** |
| **K. Yoneoka 956** | **Sporophyte** | **A** | **Nanami-sawa, Izu Oshima Island, the Izu islands, Tokyo Pref., Japan** |
| **K. Yoneoka 957** | **Sporophyte** | **A** | **Nanami-sawa, Izu Oshima Island, the Izu islands, Tokyo Pref., Japan** |
| **K. Yoneoka 958** | **Sporophyte** | **A** | **Nanami-sawa, Izu Oshima Island, the Izu islands, Tokyo Pref., Japan** |
| **K. Yoneoka 959** | **Sporophyte** | **A** | **Nanami-sawa, Izu Oshima Island, the Izu islands, Tokyo Pref., Japan** |
| **K. Yoneoka 961** | **Sporophyte** | **A** | **Nanami-sawa, Izu Oshima Island, the Izu islands, Tokyo Pref., Japan** |
| **K. Yoneoka 962** | **Sporophyte** | **A** | **Nanami-sawa, Izu Oshima Island, the Izu islands, Tokyo Pref., Japan** |
| **K. Yoneoka 963** | **Sporophyte** | **A** | **Nanami-sawa, Izu Oshima Island, the Izu islands, Tokyo Pref., Japan** |
| **K. Yoneoka 985** | **Sporophyte** | **B** | **Mt. Higashiyama, Hachijojima Island, the Izu islands, Tokyo Pref., Japan** |
| **K. Yoneoka 987** | **Sporophyte** | **B** | **Mt. Higashiyama, Hachijojima Island, the Izu islands, Tokyo Pref., Japan** |
| **K. Yoneoka 989** | **Sporophyte** | **C** | **Mt. Higashiyama, Hachijojima Island, the Izu islands, Tokyo Pref., Japan** |

**Table S3.** Records of systematic sampling. The number of "Section No." indicate the distance (m) from the starting point of the plot. Sporophyte and gametophyte presence was confirmed, with 1 indicating presence and 0 indicating absence.

| **Plot_name** | **Section No. (m)** | **Sporophytes** | **Gametophytes** |
| --- | --- | --- | --- |
| Nanami-sawa (plot A) | 0 | 1 | 1 |
| Nanami-sawa (plot A) | 1 | 0 | 1 |
| Nanami-sawa (plot A) | 2 | 0 | 1 |
| Nanami-sawa (plot A) | 3 | 0 | 1 |
| Nanami-sawa (plot A) | 4 | 0 | 1 |
| Nanami-sawa (plot A) | 5 | 1 | 0 |
| Nanami-sawa (plot A) | 6 | 1 | 0 |
| Nanami-sawa (plot A) | 7 | 0 | 1 |
| Nanami-sawa (plot A) | 8 | 0 | 1 |
| Nanami-sawa (plot A) | 9 | 0 | 1 |
| Nanami-sawa (plot A) | 10 | 0 | 1 |
| Nanami-sawa (plot A) | 11 | 0 | 1 |
| Nanami-sawa (plot A) | 12 | 0 | 1 |
| Nanami-sawa (plot A) | 13 | 0 | 1 |
| Nanami-sawa (plot A) | 14 | 0 | 1 |
| Nanami-sawa (plot A) | 15 | 0 | 0 |
| Nanami-sawa (plot A) | 16 | 0 | 0 |
| Nanami-sawa (plot A) | 17 | 0 | 0 |
| Nanami-sawa (plot A) | 18 | 0 | 0 |
| Nanami-sawa (plot A) | 19 | 0 | 0 |
| Nanami-sawa (plot A) | 20 | 0 | 0 |
| Nanami-sawa (plot A) | 21 | 0 | 0 |
| Nanami-sawa (plot A) | 22 | 0 | 1 |
| Nanami-sawa (plot A) | 23 | 0 | 1 |
| Nanami-sawa (plot A) | 24 | 0 | 1 |
| Nanami-sawa (plot A) | 25 | 0 | 1 |
| Nanami-sawa (plot A) | 26 | 1 | 0 |
| Nanami-sawa (plot A) | 27 | 1 | 0 |
| Nanami-sawa (plot A) | 28 | 1 | 1 |
| Nanami-sawa (plot A) | 29 | 1 | 0 |
| Nanami-sawa (plot A) | 30 | 0 | 1 |
| Nanami-sawa (plot A) | 31 | 0 | 1 |
| Nanami-sawa (plot A) | 32 | 0 | 1 |
| Nanami-sawa (plot A) | 33 | 0 | 1 |
| Nanami-sawa (plot A) | 34 | 0 | 1 |
| Nanami-sawa (plot A) | 35 | 1 | 1 |
| Nanami-sawa (plot A) | 36 | 1 | 1 |
| Nanami-sawa (plot A) | 37 | 1 | 1 |
| Nanami-sawa (plot A) | 38 | 0 | 0 |
| Nanami-sawa (plot A) | 39 | 0 | 1 |
| Nanami-sawa (plot A) | 40 | 0 | 1 |
| Nanami-sawa (plot A) | 41 | 1 | 0 |
| Nanami-sawa (plot A) | 42 | 0 | 1 |
| Nanami-sawa (plot A) | 43 | 0 | 0 |
| Nanami-sawa (plot A) | 44 | 0 | 0 |
| Nanami-sawa (plot A) | 45 | 1 | 0 |
| Nanami-sawa (plot A) | 46 | 1 | 0 |
| Nanami-sawa (plot A) | 47 | 1 | 0 |
| Nanami-sawa (plot A) | 48 | 1 | 0 |
| Shikamaga-taki (plot B) | 0 | 0 | 1 |
| Shikamaga-taki (plot B) | 1 | 0 | 1 |
| Shikamaga-taki (plot B) | 2 | 0 | 1 |
| Shikamaga-taki (plot B) | 3 | 0 | 1 |
| Shikamaga-taki (plot B) | 4 | 0 | 1 |
| Shikamaga-taki (plot B) | 5 | 0 | 1 |
| Shikamaga-taki (plot B) | 6 | 0 | 1 |
| Shikamaga-taki (plot B) | 7 | 0 | 1 |
| Shikamaga-taki (plot B) | 8 | 0 | 1 |
| Shikamaga-taki (plot B) | 9 | 0 | 1 |
| Shikamaga-taki (plot B) | 10 | 0 | 1 |
| Shikamaga-taki (plot B) | 11 | 0 | 1 |
| Shikamaga-taki (plot B) | 12 | 0 | 1 |
| Shikamaga-taki (plot B) | 13 | 0 | 1 |
| Shikamaga-taki (plot B) | 14 | 0 | 0 |
| Shikamaga-taki (plot B) | 15 | 0 | 1 |
| Shikamaga-taki (plot B) | 16 | 0 | 0 |
| Shikamaga-taki (plot B) | 17 | 0 | 0 |
| Shikamaga-taki (plot B) | 18 | 0 | 1 |
| Shikamaga-taki (plot B) | 19 | 0 | 0 |
| Shikamaga-taki (plot B) | 20 | 0 | 0 |
| Shikamaga-taki (plot B) | 21 | 0 | 1 |
| Shikamaga-taki (plot B) | 22 | 0 | 1 |
| Shikamaga-taki (plot B) | 21 | 0 | 0 |
| Shikamaga-taki (plot B) | 22 | 0 | 0 |
| Shikamaga-taki (plot B) | 23 | 0 | 1 |
| Shikamaga-taki (plot B) | 24 | 0 | 0 |
| Shikamaga-taki (plot B) | 25 | 0 | 0 |
| Shikamaga-taki (plot B) | 26 | 0 | 0 |
| Shikamaga-taki (plot B) | 27 | 0 | 0 |
| Shikamaga-taki (plot B) | 28 | 0 | 0 |
| Shikamaga-taki (plot B) | 29 | 0 | 0 |
| Shikamaga-taki (plot B) | 30 | 0 | 0 |
| Shikamaga-taki (plot B) | 31 | 0 | 0 |
| Shikamaga-taki (plot B) | 32 | 0 | 0 |
| Shikamaga-taki (plot B) | 33 | 0 | 0 |
| Shikamaga-taki (plot B) | 34 | 0 | 0 |
| Shikamaga-taki (plot B) | 35 | 0 | 0 |
| Shikamaga-taki (plot B) | 36 | 0 | 0 |
| Shikamaga-taki (plot B) | 37 | 0 | 1 |
| Shikamaga-taki (plot B) | 38 | 0 | 1 |
| Shikamaga-taki (plot B) | 39 | 0 | 1 |
| Shikamaga-taki (plot B) | 40 | 0 | 1 |
| Shikamaga-taki (plot B) | 41 | 0 | 0 |
| Shikamaga-taki (plot B) | 42 | 0 | 0 |
| Shikamaga-taki (plot B) | 43 | 0 | 1 |
| Shikamaga-taki (plot B) | 44 | 0 | 1 |
| Shikamaga-taki (plot B) | 45 | 0 | 1 |
| Shikamaga-taki (plot B) | 46 | 0 | 1 |
| Shikamaga-taki (plot B) | 47 | 0 | 1 |
| Shikamaga-taki (plot B) | 48 | 0 | 1 |
| Shikamaga-taki (plot B) | 49 | 0 | 0 |
| Shikamaga-taki (plot B) | 50 | 0 | 1 |
